# Structure of the perception of health professionals regarding the quality of health services in the context of COVID‐19

**DOI:** 10.1002/brb3.3140

**Published:** 2023-07-03

**Authors:** Johann M. Vega‐Dienstmaier, Alberto Fernández‐Arana, Adriel Olórtegui‐Yzú

**Affiliations:** ^1^ Facultad de Medicina Alberto Hurtado Universidad Peruana Cayetano Heredia Lima Peru; ^2^ Instituto de Neurociencias Aplicadas Lima Perú; ^3^ Facultad de Medina de San Fernando Universidad Nacional Mayor de San Marcos Lima Peru; ^4^ Instituto Nacional Cardiovascular—INCOR—EsSalud Lima Peru

**Keywords:** COVID‐19, health professionals, health services, network analysis

## Abstract

**Background:**

The health emergency caused by COVID‐19 revealed the shortcomings of health services (HS), but little is known about how this has impacted the mental health of health professionals (HP) when perceiving these difficulties.

**Methods:**

Data were collected through an online survey administered to HP in Lima (Peru) between May and July 2020. A questionnaire was applied to identify perceived quality of health services (PHQS). A network analysis was performed, and the centrality measures of the variables were calculated and plotted.

**Results:**

A total of 507 HP completed the survey. In the network analysis of PHQS, four clusters were identified: (A) “empathy” and “recognition of competencies,” (B) “logistical support,” “protection,” “personal early diagnosis,” and “early family diagnosis”; C) “professional competence with regard to their treatment and treatment for their family,” “equipment for their treatment and treatment for their family,” “professional competence with regard to their treatment and treatment for their family,” and “institutional support for them and their family”; and D) “fear of being infected or infecting their family,” “fear of dying or death of a family member,” “knowledge stability,” “job burnout,” and “role change.” The variables of PHQS with the greatest centrality were “equipment for their treatment,” “equipment for the treatment of their family,” and “early family diagnosis.”

**Conclusions:**

The structure of the PHQS of HP describes direct and indirect influences of different variables in the context of COVID‐19.

## Graphical abstract

The health emergency created by the COVID‐19 pandemic has put the solvency of health services to the test. Health professionals may be affected by the perception of precariousness of health services. These perceptions could affect the adaptability and resilience of health professionals in the face of stress, such as the COVID‐19 pandemic.

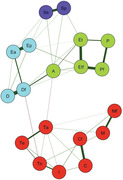

## 1 INTRODUCTION

The health emergency created by the COVID‐19 pandemic has put the solvency of health services (HS) to the test, regarding not only their ability to face the growing demand for patient care but also in the availability of qualified health professionals (HP), technological means of diagnosis and efficient treatment, medical supplies for the adequate care of patients, and optimal working conditions for HP (Chen et al., 2020; Organización Panamericana de la Salud, 2022).

At the time of writing this article, Peru registered 4,330,521 infections and 217,566 deaths from COVID‐19 (World Health Organization, 2021), with 2305 cases of infection in HP (Centro Nacional de Epidemiología, Prevención y Control de Enfermedades, s. f.). Peru adopted the health recommendations against the pandemic issued by the World Health Organization (WHO) early; therefore, the high numbers of infections and deaths reported imply a lack of efficacy of these strategies (Díaz Cassou et al., 2020); the general population had difficulty complying with the measures because of the precariousness, inequity, and fragmentation of HS, an economy whose informal sector supports 70% of the national economy (PERUCÁMARAS: Cámara Nacional de Comercio, Producción, Turismo y Servicios, 2020), families living in overcrowded conditions (11.7%), and households without hygienic services (29.2% for metropolitan Lima alone) (Instituto Nacional de Estadística e Informática, 2020).

For HP, the extraordinary conditions in which they worked during the pandemic were confounding factors, for example, increased workloads, shortages of drugs or specific vaccines, a lack of personal protective equipment, and the feeling of not receiving enough support from health authorities (Cuba, 2021). This context, added to the impact on quality of life, explains why the mental health of the HP who faced COVID‐19 on the front line (Pearman et al., 2020; Shanafelt et al., 2020; Vizheh et al., 2020) was affected, leading to a deterioration in their clinical understanding capacities and/or decision‐making skills (Siddiqui et al., 2021). Consequently, the COVID‐19 pandemic must be considered a powerful and persistent stressor, whose effects on the mental health of HP stem from (Ambrosetti et al., 2021; Amerio et al., 2021; Nobari et al., 2021) among other factors, the discomfort experienced as a result of precarious working conditions and feelings of being abandoned by health authorities who did not comply with the duty to protect them (Charney et al., 2020; Crocker et al., 2023; Editorial eClinicalMedicine, 2020; Fernández‐Arana et al., 2022; Lin et al., 2020; Olórtegui‐Yzú et al., 2023; Organización Panamericana de la Salud, 2022; Young et al., 2021).

This study is part of a project aimed to evaluate various aspects of the mental health of HP during the pandemic, developed in the city of Lima, Peru; some of the findings of the project have been recently published. In the first study, the relationship between mental health problems (anxiety, depression, and perceived stress) and the care of patients with COVID‐19 and early adverse events (ACEs) in HP was evaluated (Fernández‐Arana et al., 2022). A total of 542 health professionals completed the survey. Caring for patients with COVID‐19 was significantly associated with depression and anxiety. Moreover, when caring for patients with COVID‐19 was combined with a history of early sexual abuse, its effect on the risk of anxiety increased (OR = 7.71, *p* = .010). Mental health problems were associated with female gender in almost all the analyses and with most ACEs. In a second study carried out with the same HP population, the relationship between mental health problems (anxiety, depression, and PS) and their perceptions about the quality of health services (PQHS) in the context of the COVID‐19 pandemic was established (Olórtegui‐Yzú et al., 2023). The most relevant unfavorable PQHS associated with anxiety were “HP competence with regard to their treatment”, as well as “institutional support for themselves and their families” in the event of becoming infected; the most relevant unfavorable PHQS associated with depression were “equipment for their treatment and their families” if infected, “institutional support for themselves and their families” if infected, “fear of HP and/or family menbers being infected or dying”, and “HP' recognition of their competencies” (*p* < .001); and most relevant unfavorable PHQS associated with PS were “institutional support for themselves and their families” if infected and “inestability of knowldge”.

The objective of this study was to establish, through a network analysis, the structure of the relationships between the PHQS by HP in the context of the COVID‐19 pandemic.

## 2 MATERIALS AND METHODS

A cross‐sectional survey was administered to 507 HP without a prior diagnosis of COVID‐19 in Lima, Peru. The selection of professionals was by invitation. The sample size was calculated using Epidat v 3.1 (Consellería de Sanidade—Servizo Galego de Saúde, s. f.), considering maximum variability, a significance of 0.05, a precision of 5%, and a data loss of 10%. The HP included were physicians, nurses, midwives, medical technologists, nutritionists, pharmaceutical chemists, nursing technicians, psychologists, and biologists. Considering that no professional distinction was made regarding front‐line care due to the lack of personnel for the identification and care of patients with COVID‐19, health institutions assigned all available health professionals to work directly or indirectly with COVID patients.

Personal, work, health, and mental health service variables were measured through a questionnaire containing 48 questions prepared with Google Forms® and distributed online during the months of May and June 2020. The questionnaire was anonymous to ensure the privacy and confidentiality of the participants. The structure of the survey did not allow the next question to be presented until the previous question was answered.

The PHQS of HP were evaluated by means of 19 questions, corresponding to the same number of dichotomous variables (“favorable perception” = 1 or “unfavorable perception” = 0). The PHQS variables covered five main themes: (i) recognition of the concerns and competencies of HP; (ii) protection of HP and their families against the spread of SARS‐COV‐2; (iii) solvency of HSs with regard to the treatment of HP and their families in case of infection; (iv) fear of infection and death of HP and/or their family members due to SARS‐COV‐2; and (v) optimization of the work performance of HP (see Table 1). This questionnaire was developed by the authors based on other studies on this topic (Charney et al., 2020; Crocker et al., 2023; Editorial eClinicalMedicine, 2020; Lin et al., 2020).

**TABLE 1:** Grouping and assignment of variables of perceived quality of health services (PHQS) of health professionals (HP) in the context of the pandemic.

PHQS	Direct variable	Abbrev	Questions
Recognition of the concerns and competencies of HP	Empathy	**Sp**	Health authorities recognize and understand my concerns
	Recognition of competencies	**Se**	Health authorities recognize my experience
Protection of HP and their families against the spread of SARS‐COV‐2	Protection	**Ep**	I receive the appropriate protective equipment for this pandemic
	Logistic support	**Ea**	I have the necessary equipment to care for my infected patients
	Early diagnosis	**D**	I have quick access to diagnostic systems to know if I am infected by the virus
	Family early diagnosis	**Df**	I have quick access to diagnostic systems to know if my family is infected with the virus
Solvency of HSs with regard to the treatment of HP and their families in the event of infection	Professional competence with regard to their treatment	**P**	If I got infected, I would receive adequate care from my colleagues because they are trained
	Professional competence with regard to treatment for their family	**Pf**	If a family member was infected, he or she would receive adequate care because my colleagues are trained
	Equipment for their treatment	**Et**	If I were infected, I would receive adequate care from the hospital because it has adequate equipment
	Equipment for the treatment of their family	**Etf**	If a family member was infected, he or she would receive adequate care from the hospital because the hospital has adequate equipment
	Institutional support for themselves and their families	**A**	If I got sick, I would receive support from the health authorities for myself and my family
Fear of infection and death of HP and/or their families due to SARS‐COV‐2	Fear of getting infected	**C**	I am afraid of catching it
	Fear of infecting the family	**Cf**	I am afraid of infecting my loved ones
	Fear of dying	**M**	If I got infected, I would probably die
	Fear of the death of a family member	**Mf**	If my family member was infected, he or she would probably die
Working conditions for HP	Knowledge instability	**I**	I am concerned that the information and procedures will change day by day
	Job burnout	**Te**	The work is strenuous and does not allow me to be fully effective
	Concern for the family	**Ta**	I cannot stop thinking about my family when I am at work, and that affects my concentration
	Role change	**Tn**	I am concerned about being in an area of care that is not my specialty

### 2.1 Data analysis

The network analysis was performed in RStudio® using the packages *IsingFit, qgraph*, and *igraph*. The network was estimated with the *IsingFit* function. Measures of centrality of the variables (strength and influence) were calculated and plotted using the *centralityTable* and *centralityPlot* functions. Clusters of variables were determined with the *cluster_walktrap* and *communities* function. The network with its corresponding clusters was plotted using the *qgraph* function.

### 2.2 Ethical aspects

The study procedures were carried out in accordance with the ethical standards for research in human beings, receiving approval by the Research Ethics Committee of the “Carlos Alberto Peschiera Carrillo” National Cardiovascular Institute—INCOR—EsSalud. Certificate of Approval number 09/2020‐CEI of June 14, 2020.

## 3 RESULTS

### 3.1 General

The survey was answered by 507 HP, whose average age was 49.5 years (minimum 23 years and maximum 87 years). A total of 62.7% were women, with a mean age of 47.7 years; for men, the average age was 52.5 years (*p* < 0.05) (Fernández‐Arana et al., 2022).

Doctors and nurses accounted for 56.8% and 15.0%, respectively, of the respondents, followed by obstetric professionals (11.6%). These three groups accounted for 88.4% of the total respondents. Married professionals or those cohabiting accounted for 59.5% of the respondents. A total of 38.3% of those surveyed worked directly with patients with COVID‐19 (Fernández‐Arana et al., 2022).

Regarding the PHQS of HP not infected with COVID‐19, the two items most frequently reported were “fear of infecting their family” and “fear of being infected” (92.5% and 89.0%, respectively). These items were followed in frequency by “knowledge instability” and “fear of a family member dying” (80.5% and 72.0%, respectively). Notably, 69.0% reported “job burnout” (Olórtegui‐Yzú et al., 2023).

### 3.2 Network analysis

The associations between the variables corresponding to PHQS and from which the network analysis was performed are shown in Table 2.

**TABLE 2:** Association matrix of the estimated network of the perceived quality of health services (PHQS) of health professionals (HP).

	Sp	Se	Ep	D	Df	Ea	C	P	Et	M	Cf	Pf	Etf	Mf	Tn	I	Te	Ta	A
**Sp**		2.18	0.24	0.00	0.00	0.00	0.00	0.00	0.42	0.00	0.00	0.00	0.00	0.00	0.00	0.00	0.00	0.00	0.42
**Se**	2.18		0.29	0.00	0.00	0.29	0.00	0.00	0.00	0.00	0.00	0.00	0.00	0.00	0.00	0.00	0.00	0.00	0.47
**Ep**	0.24	0.29		0.63	0.00	0.36	0.00	0.00	0.00	0.00	0.00	0.00	0.70	0.00	0.00	0.00	0.00	0.00	0.00
**D**	0.00	0.00	0.63		3.06	1.19	0.00	0.00	0.00	0.00	0.00	0.00	0.00	0.00	0.00	0.00	0.00	0.00	0.15
**Df**	0.00	0.00	0.00	3.06		0.00	0.00	0.00	0.34	0.00	0.00	0.00	0.27	0.00	‐0.46	0.00	0.00	0.39	1.11
**Ea**	0.00	0.29	2.71	0.36	1.19		0.00	0.00	0.12	0.00	0.00	0.00	0.00	0.00	0.00	0.00	0.00	0.00	0.00
**C**	0.00	0.00	0.00	0.00	0.00	0.00		0.00	0.00	0.00	2.07	0.00	0.00	0.00	0.00	0.57	0.00	0.39	0.00
**P**	0.00	0.00	0.00	0.00	0.00	0.12	0.00		1.11	0.00	0.00	1.87	0.00	0.00	0.00	0.00	0.00	0.00	0.00
**Et**	0.42	0.00	0.00	0.15	0.34	0.00	0.00	1.11		0.00	0.00	0.00	2.31	0.00	0.00	0.00	0.00	0.00	0.83
**M**	0.00	0.00	0.00	0.00	0.00	0.00	0.00	0.00	0.00		0.00	0.00	0.00	1.77	0.51	0.00	0.00	0.37	0.00
**Cf**	0.00	0.00	0.00	0.00	0.00	0.00	2.07	0.00	0.00	0.00		0.00	0.00	0.64	0.00	0.80	0.00	0.00	0.00
**Pf**	0.00	0.00	0.00	0.00	0.00	0.00	0.00	1.87	0.00	0.00	0.00		2.33	0.00	0.00	0.00	0.00	0.00	0.26
**Etf**	0.00	0.00	0.00	0.00	0.27	0.00	0.00	0.00	2.31	0.00	0.00	2.33		0.00	0.00	0.00	0.00	0.00	0.74
**Mf**	0.00	0.00	0.68	0.00	0.00	0.00	0.00	0.00	0.00	1.77	0.64	0.00	0.00		0.00	0.00	0.00	0.18	0.00
**Tn**	0.00	0.00	0.00	0.00	‐0.46	0.00	0.00	0.00	0.00	0.51	0.00	0.00	0.00	0.00		1.22	0.88	0.35	0.00
**I**	0.00	0.00	0.00	0.00	0.00	0.00	0.57	0.00	0.00	0.00	0.80	0.00	0.00	0.00	1.22		0.59	0.34	0.00
**Te**	0.00	0.00	0.00	0.00	0.00	0.00	0.00	0.00	0.00	0.00	0.00	0.00	0.00	0.00	0.88	0.59		1.49	0.00
**Ta**	0.00	0.00	0.00	0.00	0.00	0.00	0.39	0.00	0.00	0.37	0.00	0.00	0.00	0.18	0.35	0.34	1.49		‐0.31
**A**	0.42	0.47	0.00	0.05	1.11	0.00	0.00	0.00	0.83	0.00	0.00	0.74	0.74	0.00	0.00	0.00	0.00	‐0.31	

Sp, empathy; Se, recognition of competencies; Ep, protection; D, early diagnosis; Df, early family diagnosis; Ea, logistical support; P, professional competence with regard to their treatment; Et, equipment for their treatment; Pf, professional competence with regard to treatment for their family; Etf, equipment for the treatment of their family; Cf, fear of infecting their family; C, fear of getting infected; M, fear of dying; Mf, fear of a family member dying; I, knowledge stability; Te, job burnout; Ta, concern for the family; A, institutional support for themselves and their family; Tn, role change.

**FIGURE 1:**
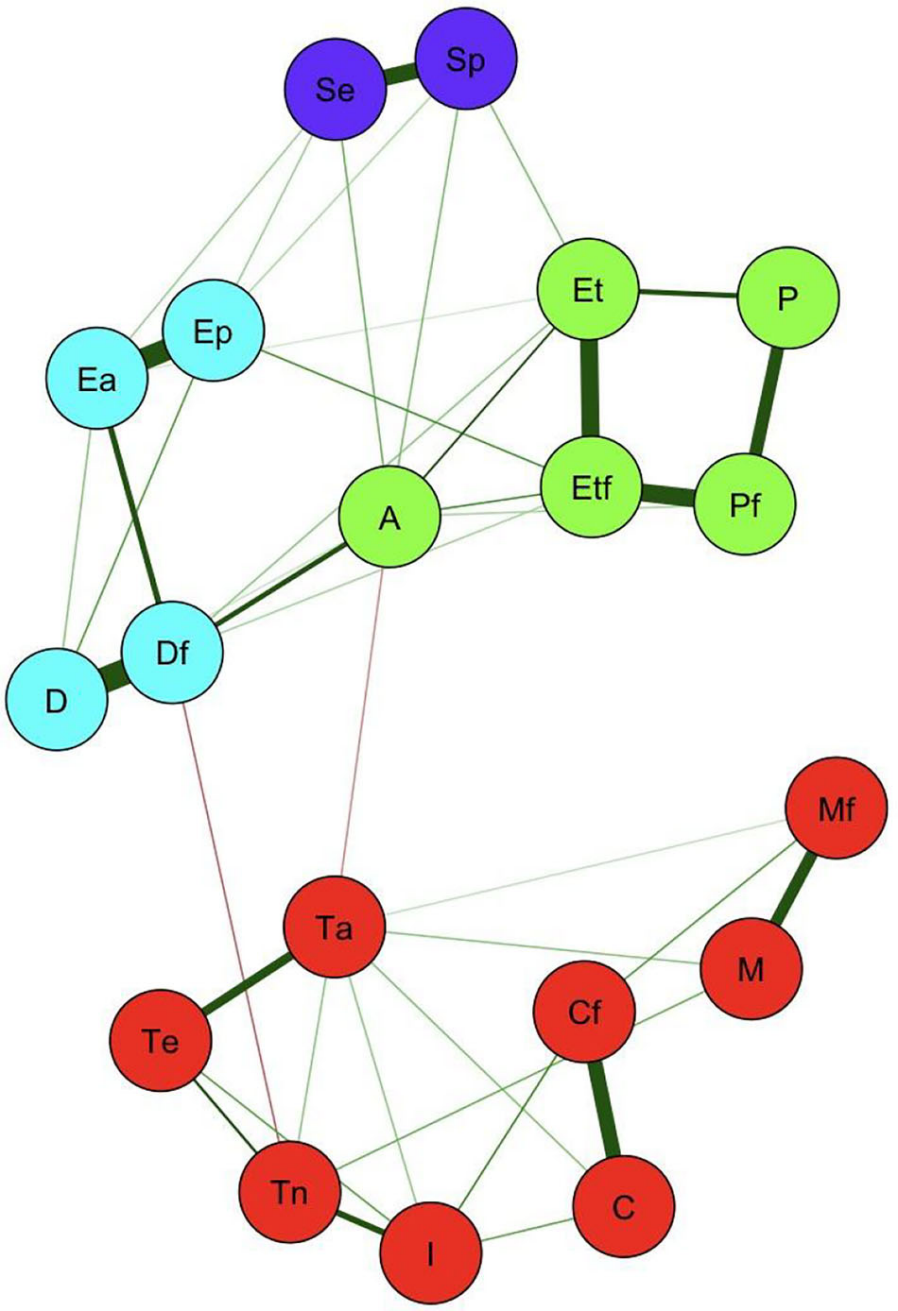
Network of associations of the perceived quality of health services (PHQS) of health professionals (HP). Cluster A (purple): Sp, empathy; Se, recognition of competencies. Cluster B (light blue): Ep, protection; Ea, logistical support; D, early diagnosis; Df, early family diagnosis. Cluster C (green): P, professional competence with regard to their treatment; Et, equipment for their treatment; Pf, professional competence with regard to treatment for their family; Etf, equipment for the treatment of their family; A, institutional support for themselves and their family. Cluster D (in red): Cf, fear of infecting the family; C, fear of getting infected; M, fear of dying; Mf, fear of family member dying; I: knowledge stability; Te, job burnout; Ta, concern for the family; Tn, role change. Green lines: positive correlations; red lines: negative correlations.

In the network analysis of the PHQS, four clusters were identified, characterized by the following themes: (A) empathy and recognition of competencies (in purple); (B) logistical support, protection, personal and family early diagnosisʺ (light blue); (C) professional competence and equipment for treating HP and treating their families and institutional support for HP and their families (green); and (D) fear of being infected or infecting family members, fear of dying or fear of a family member dying, concern for the family, knowledge instability, job burnout, and role changes (red) (Figure 1).

**FIGURE 2:**
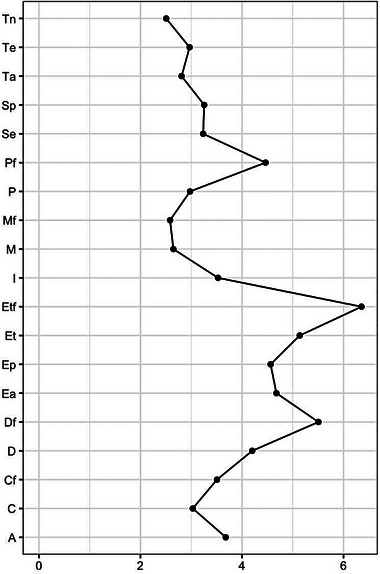
Measures of centrality (influence) for each perceived quality of health services (PHS) of HCPs. Sp, empathy; Se, recognition of competences; Ep, protection; D, early diagnosis; Df, early family diagnosis; Ea, logistical support; P, professional competence with regard to their treatment; Et, equipment for their treatment; Pf, professional competence with regard to treatment for their family; Etf, equipment for the treatment of their family; Cf, fear of infecting the family; C, fear of getting infected; M, fear of dying; Mf, fear of family member dying; I, knowledge stability; Te, job burnout; Ta, concern for the family; A, institutional support for themselves and their family; Tn, role change.

**FIGURE 3:**
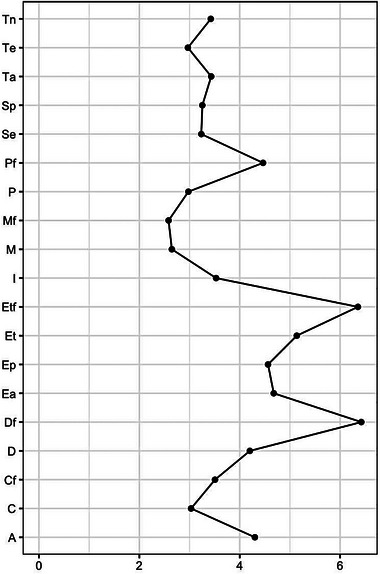
FIGURE 3 Measures of centrality (strength) for each perceived quality of health services (PHS) of HCPs. Sp, empathy; Se, recognition of competencies; Ep, protection; D, early diagnosis; Df, early family diagnosis; Ea, logistical support; P, professional competence with regard to their treatment; Et, equipment for their treatment; Pf, professional competence with regard to treatment for their family; Etf, equipment for the treatment of their family; Cf, fear of infecting the family; C, fear of getting infected; M, fear of dying; Mf, fear of family member dying; I, knowledge stability; Te, job burnout; Ta, concern for the family; A, institutional support for themselves and their family; Tn, role change.

The centrality measures for each PHS variable are graphed in Figure 2 (influence) and Figure 3 (strength). Those variables with the greatest centrality were “equipment for treating family members,” “early family diagnosis,” and “equipment for treating HP.”

## 4 DISCUSSION

In the network analysis of the PHQS of HP, four clusters were identified, three of which coincided with those proposed by the authors: (A) recognition of the concerns and competencies of HP, consisting of the variables “empathy” and “recognition of competencies”; (B) protection of HP and their families against infection by the virus, consisting of the variables “logistical support,” “protection,” “early personal diagnosis,” and “early family diagnosis”; and (C) solvency of HSs with regard to the treatment of HP and their family members in case of infection, which includes professional competence with regard to treating HP and their family members, the equipment for treating HP and their family members, professional competence with regard to treating HP and their family members, and institutional support for HP and their family. The fourth cluster (D) included two categories proposed by the authors: (1) fear of infection and death of HP and/or their family members and (2) the conditions in which HP work, which in turn involves knowledge instability, job burnout, impacts on work due to concerns for their family, and role changes (Table 1 and Figure 1).

Cluster A shows the importance of HS authorities being attentive and recognizing the abilities and concerns of HP, a result that aligns with the findings of a study carried out in a university hospital in Rome (Magnavita et al., 2020), where it was reported that anesthesiologists directly involved in the care of patients with suspected or confirmed cases of COVID‐19 perceived a low level of recognition or consideration from the authorities regarding their professional experience and how it can influence and/or modify the outcomes of procedures. Likewise, Cluster A is closely linked to the concerns of HP regarding resources for protection against infection (Cluster B) and the possibility of accessing adequate treatment for HP and their family if they become ill (Cluster C) (Figure 1).

Cluster B shows the importance of HSs providing elements for protection against infection necessary for HP to care for patients and to avoid infecting their families. In line with our findings, a study in Bangladesh showed the difficulties HP encountered performing their duties while wearing inadequate personal protective equipment (PPE) in hospitals. In addition, they had to meet their family responsibilities despite the risk of infection (Mehedi & Ismail Hossain, 2022). Another study carried out in the United Kingdom (Nyashanu et al., 2020) highlights how the shortage of PPE prevented HP from being able to fully comply with their hospital duties and deteriorated relationships with colleagues. In addition, these perceptions represented by Cluster B are strongly linked to the confidence that HSs will provide early diagnosis services for both HP and their families (strongly connected perceptions). This group was directly related to the perceptions that address the solvency of HSs to offer HP and their families adequate and efficient treatment in the event they are infected with COVID‐19; in addition, it affected HCP trust in receiving support from HSs if they or a family member died as a result of COVID‐19 (Cluster C), (Figure 1).

Cluster C reveals the importance of perceiving the solvency of HSs about the treatment of HP and their families in the event of infection, from the points of view of both the competency of the professionals and available equipment (mechanical respirators, oxygen therapy, etc.). In Peru, as in other countries, hospitals had to convert operating rooms and general rooms into intensive care units (ICUs) and use anesthetic ventilators, portable ventilators, and older ventilators from laboratories or teaching simulation areas. However, in many hospitals, these resources were quickly depleted, as was the supply of oxygen (Pons‐Òdena et al., 2020). In these circumstances, physicians must decide whether to allocate or reallocate these resources during shortages (Chu et al., 2020). This explains why HP, given this shortage of ventilators and oxygen, perceived that they and their families will not receive adequate care if they are infected. In addition, the constant change in and adaptation of equipment made it difficult to adequately train colleagues on correct use (Chu et al., 2020; Pons‐Òdena et al., 2020). On this basis, it is understandable why the perceptions of HP regarding “equipment for their treatment,” “equipment for their family,” and “early family diagnosis” are those that in the analysis of centrality have the greatest strength and influence (Figures 2 and 3). A study carried out in Iran described that the concern of HP for their health and the health of their relatives, in particular elderly individuals, was directly related to the need to comply with caring for patients with minimal PPE in hospitals (Eftekhar Ardebili et al., 2021).

Cluster D shows the importance of the perceptions of HP in relation to the fear of infection and subsequent death of themselves and/or their relatives and their work performance. The influence of “concern for the family” is highlighted as a relevant factor that affects concentration at work and has a close relationship with job burnout and concern about providing services outside of their role to meet HS needs. These factors were coupled with an instability of information that constantly changes and a lack of real knowledge about the behavior of COVID‐19. These findings coincide with the results of a study in Turkey (Alsulimani et al., 2021) that reported that 46% of the HP studied had job burnout closely related to the perception of being pressured to work in patient care services due to COVID‐19. Another study in Bangladesh (Hossain et al., 2021) concluded that the shortage of PPE and the lack of training to combat COVID‐19 were among the leading reasons that generated a fear of infection and death of HP and their families. These conditions, added to failures in HS management, generated a decrease in HCP competence. A study in the United Kingdom (Vindrola‐Padros et al., 2020) reported that frequent changes in the guidelines for coping with the diagnosis and treatment of COVID‐19 infection caused excessive pressure on exposed personnel, generating a decrease in the effectiveness of their work performance. An investigation that included 60 countries also identified that there was a relationship of workload and inadequate organizational support with burnout among HP (Morgantini et al., 2020). Finally, an investigation in China (Chang et al., 2020) observed that the fear of infection in HP traumatized them, preventing them from carrying out their work in a solvent manner.

This study has several limitations. First, it was a cross‐sectional study based on an online questionnaire distributed to HP. Generalization of the conclusions is not possible because we did not have a means of approximating the entire population of HP. A longitudinal design is needed to provide definitive evidence of the resilience of health workers against mental health problems related to COVID‐19. Second, the participants were heterogeneous and direct conclusions could not be applied to any professional group, whether they were doctors, nurses, midwives, or others. Third, the participants were from Lima and the surrounding region; therefore, our findings cannot be generalized to the less affected regions of Peru or to professionals from other countries due to cultural differences and differences in health systems. Fourth, this study was based on a self‐administered questionnaire, and it was not possible to verify mental health problems through structured interviews. Fifth, this study did not differentiate between preexisting mental health symptoms and new symptoms related to COVID‐19 care.

## 5 CONCLUSIONS

The results of this study, beyond the understandable effects on the mental health of HP in the context of a health emergency such as the COVID‐19 pandemic, highlight the importance of PHQS related to the precariousness of HSs, both in budgetary as well as administrative, technological, and assistance aspects, which could affect the adaptability and resilience of workers to these contingencies.

The network analysis shows us how the PHQS of the HP are grouped, related, and hierarchical, allowing us to identify the qualitative impact of each subgroup on their adaptability and efficiency in their job performance, in the context of the COVID‐19 pandemic.

Therefore, it is necessary for HP to be able to express their concerns in a systematic and ongoing way regarding how HSs are fulfilling their responsibilities of leadership, protection, and support of HP and their families in general and especially in health emergency situations like COVID‐19. Only in this way can effective individual and group psychotherapeutic interventions be implemented consistently. In addition, HS authorities will have greater tools for proposing tangible changes to governments to solve these needs.

## AUTHOR CONTRIBUTIONS

**Johann M. Vega‐Dienstmaier**: Collaborated in the elaboration of the survey, performed the computations, and contributed to the interpretation of the results. **Alberto Fernández‐Arana**: Conceived the presented idea, developed the theory, collaborated in the elaboration of the survey, and contributed to the interpretation of results. **Adriel Olórtegui‐Yzú**: Collaborated in the elaboration of the survey, performed the computations, and contributed to the interpretation of the results.

## CONFLICT OF INTEREST STATEMENT

The authors declare no conflicts of interest.

### PEER REVIEW

The peer review history for this article is available at https://publons.com/publon/10.1002/brb3.3140

## Data Availability

Data available on request from the authors.
